# Cell Deformation at the Air-Liquid Interface Evokes Intracellular Ca^2+^ Increase and ATP Release in Cultured Rat Urothelial Cells

**DOI:** 10.3389/fphys.2021.631022

**Published:** 2021-02-03

**Authors:** Jiliang Wen, Zhenghao Chen, Mengmeng Zhao, Shulu Zu, Shengtian Zhao, Shaoyong Wang, Xiulin Zhang

**Affiliations:** ^1^Department of Urology, The Second Hospital, Cheeloo College of Medicine, Shandong University, Jinan, China; ^2^Department of Urology, Shandong Provincial Hospital, Cheeloo College of Medicine, Shandong University, Jinan, China

**Keywords:** urothelial cells, TRPV4 channel, pannaxin-1 channel, mechanosensory transduction, ATP

## Abstract

Urothelial cells have been implicated in bladder mechanosensory transduction, and thus, initiation of the micturition reflex. Cell deformation caused by tension forces at an air-liquid interface (ALI) can induce an increase in intracellular Ca^2+^ concentration ([Ca^2+^]_i_) and ATP release in some epithelial cells. In this study, we aimed to examine the cellular mechanisms underlying ALI-induced [Ca^2+^]_i_ increase in cultured urothelial cells. The ALI was created by stopping the influx of the perfusion but maintaining efflux. The [Ca^2+^]_i_ increase was measured using the Ca^2+^ imaging method. The ALI evoked a reversible [Ca^2+^]_i_ increase and ATP release in urothelial cells, which was almost abolished by GdCl_3_. The specific antagonist of the transient receptor potential vanilloid (TRPV4) channel (HC0674) and the antagonist of the pannexin 1 channel (^10^panx) both diminished the [Ca^2+^]_i_ increase. The blocker of Ca^2+^-ATPase pumps on the endoplasmic reticulum (thapsigargin), the IP3 receptor antagonist (Xest-C), and the ryanodine receptor antagonist (ryanodine) all attenuated the [Ca^2+^]_i_ increase. Degrading extracellular ATP with apyrase or blocking ATP receptors (P2X or P2Y) with pyridoxalphosphate-6-azophenyl-2',4'-disulfonic acid (PPADS) significantly attenuated the [Ca^2+^]_i_ increase. Our results suggest that both Ca^2+^ influx *via* TRPV4 or pannexin 1 and Ca^2+^ release from intracellular Ca^2+^ stores *via* IP3 or ryanodine receptors contribute to the mechanical responses of urothelial cells. The release of ATP further enhances the [Ca^2+^]_i_ increase by activating P2X and P2Y receptors *via* autocrine or paracrine mechanisms.

## Introduction

The urothelium of the bladder has been previously considered a passive barrier. However, in recent years, a large number of reports have shown that the urothelium plays an important role in bladder mechanosensory transduction (de Groat, 2004; Birder, 2005). In response to mechanical stimuli, two critical concomitant intracellular events occur in urothelial cells: an increase in intracellular Ca^2+^ concentration ([Ca^2+^]_i_) and the release of signaling molecules such as NO, ATP, and acetylcholine (ACh). The release of ATP from urothelial cells directly activates purinergic receptors on bladder afferent terminals to increase their excitability, thereby enhancing sensory function of the bladder (Yu and de Groat, 2008). Although several studies and a few reviews have been conducted on mechanosensing in urothelial cells (Merrill et al., 2016; Janssen et al., 2017), the exact cellular mechanisms by which urothelial cells sense mechanical stimuli are not fully understood.

The activation of mechanosensitive channels (MSCs) on urothelial cells is the first step in initiating mechanotransduction. These channels detect and transduce external mechanical forces into intracellular Ca^2+^ elevation and ATP release. Most MSCs are nonselective cation channels with high Ca^2+^ permeability that contribute to the elevation of intracellular Ca^2+^. Several classes of membrane-bound MSCs have been implicated in the urothelial mechanotransduction. These channels include acid-sensing ion channels (Sadananda et al., 2009); transient receptor potential vanilloid (TRPV1 or TRPV4) channels (Birder et al., 2002; Mochizuki et al., 2009); epithelial Na^+^ channels (Du et al., 2007); the Piezo1 channel (Miyamoto et al., 2014); Piezo2 channel (Marshall et al., 2020); pannexin hemichannels (Timoteo et al., 2014; McLatchie and Fry, 2015); and connexin channels (Shimura et al., 2018).

*In vivo*, changes in intravesical pressure or bladder wall tension are physiologically relevant mechanical stimuli for the urothelium. To simulate these stimuli *in vitro*, common strategies include stretching of urothelial cells cultured on coverslips using various customized devices (Miyamoto et al., 2014; Guan et al., 2018) or exposing urothelial cells to high hydrostatic pressure (Olsen et al., 2011). For example, custom designed chambers, attached to an extension device, were used to stretch urothelial cells in one study (Miyamoto et al., 2014). In another study, urothelial cells cultured on plastic dishes were exposed to sustained hydrostatic pressure (5–20 cm H_2_O; Olsen et al., 2011).

The above approaches of delivering mechanical stimuli are valuable in revealing the underlying mechanisms of urothelial mechanotransduction. However, they require technically complicated setups. In other reports in the literature, exposure of urothelial cells to low-osmolality solutions to induce cell swelling was also a frequent strategy to alter cell membrane stress (Haeberle et al., 2008; Wu et al., 2011). Delivering hypoosmotic shock to cells is simple. However, this modality might not present a physiologically relevant stimulus. Although low-osmolarity urine is occasionally present in the bladder *in vivo*, it may not lead to the swelling of urothelial cells. This is because of the presence of specialized lipid molecules and uroplakin proteins in the apical membrane of umbrella cells, which reduce the permeability of the cells to water.

Several studies have reported that cells can be mechanically stimulated by creating an air-liquid interface (ALI). The ALI is produced by reducing the volume of liquid covering cells, and a thin film of liquid on the cells is drawn down to such proximity that tension forces at the ALI cause cell deformation (Patrick et al., 2001; Ramsingh et al., 2011). In the study by Ramsingh et al. (2011), lung epithelial cells were exposed to ALI forces by briefly introducing an air bubble into a closed perfusion chamber or by tilting the culture dish containing the cells. Such stimuli produced reversible cell deformation, a transient increase in [Ca^2+^]_i_, and ATP release. In another study, a microfluidic device was developed to create an ALI by liquid evaporation (Huang et al., 2016), to investigate ALI-induced intracellular Ca^2+^ response in a human leukemic cell line (HL-60).

In the present study, we developed another method to create an ALI in a perfusion cell chamber, by simply stopping influx, but maintaining efflux of the perfusion, until a thin layer of liquid was created on the urothelial cells. We found that the ALI can induce a remarkable increase in [Ca^2+^]_i_ and ATP release from urothelial cells cultured on glass coverslips, and the responses were repeatable and did not affect cell viability. We aimed to investigate the underlying cellular mechanisms of mechanical stimulus-induced increase in [Ca^2+^]_i_ with this easily-operated model. Our main focus was on the potential involving channels or receptors located either on the cell membrane or those associated with intracellular Ca^2+^ stores. We found that both Ca^2+^ influx *via* membrane-bound TRPV4 or pannexin 1 channels, and Ca^2+^ release from intracellular Ca^2+^ stores *via* IP3 and ryanodine receptors contribute to an ALI-induced [Ca^2+^]_i_ increase. Furthermore, ATP released from urothelial cells also contribute to a [Ca^2+^]_i_ increase *via* autocrine or paracrine mechanisms.

## Materials and Methods

### Experimental Animals

Female Sprague Dawley rats (virgins, 2–3 months old, 180–250 g; obtained from Pengyue Animal Co., Jinan, China) were used in this study. Rats were allowed free movement, and had free access to food and water. Care and handling of the animals were in accordance with the Shandong University Animal Care and Use Committee. The study was approved by the Ethics Committee of the Second Hospital, Cheeloo College of Medicine, Shandong University [KYLL-2016(GJ)A-0027].

### Urothelial Cell Culture

Urothelial cell cultures were performed as previously described (Kullmann et al., 2009). Rats were subjected to isoflurane anesthesia, and bladders were resected and placed in cold minimal essential medium (MEM, Invitrogen, Carlsbad, CA, United States) supplemented with HEPES (2.5 g/L, Sigma, St. Louis, MO, United States) and penicillin/streptomycin/fungizone (PSF, 1%, Sigma). The bladder urothelium was incubated in Dispase (2.5 mg/ml, Worthington Biochemical Lakewood, NJ, United States) overnight at 4°C. Urothelial cells were gently scraped, placed in trypsin (0.25% wt/vol, Sigma) for 10–15 min at 37°C, and dissociated by trituration. Cells were suspended in MEM containing 10% FBS and centrifuged at 416 *g* for 5 min. The cells were suspended in urothelial cell medium (UCM, ScienCell, San Diego, CA, United States) with 1% PSF, centrifuged again, and resuspended in fresh media. Cells were plated on poly-L-lysine-coated glass coverslips and used for Ca^2+^ imaging, 48–96 h after dissociation.

### Creation of the Air-Liquid Interface

**Figure 1:**
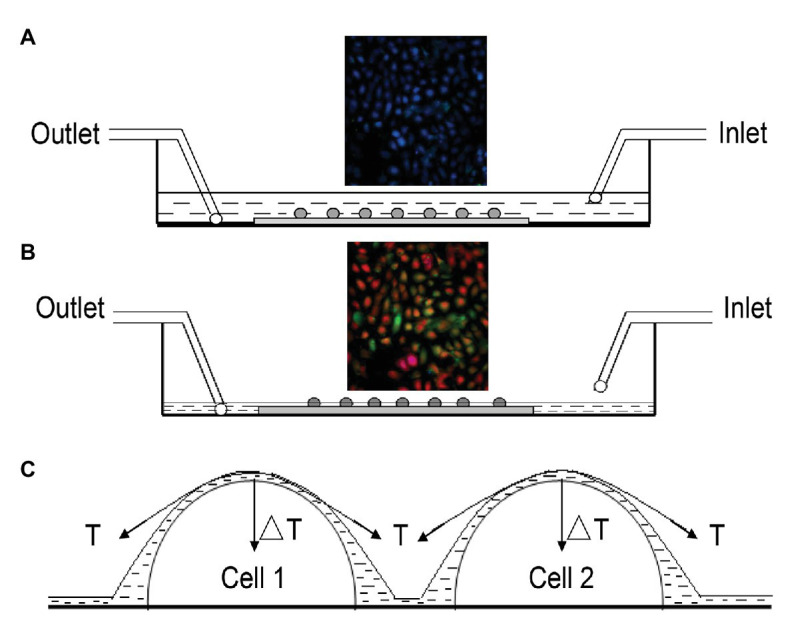
Illustration of approach to produce the air-liquid interface (ALI) on urothelial cells. **(A)** Cross-sectional view of the flow chamber continuously perfused with Hank’s balanced salt solution (HBSS), for which influx was driven by gravity and efflux was driven by a vacuum pump. Monolayer urothelial cells were immersed in HBSS, and no ALI was formed when influx and efflux were balanced. Fluorescence signals at 340 or 380 nm were monitored simultaneously, and signals showed no change (inset). **(B)** To produce the ALI, influx was stopped but efflux was continued. Once the amount of HBSS solution in the chamber was reduced to a specific level at which a thin liquid film was formed on the cells, they were mechanically deformed by the tension force at the ALI, which altered the fluorescence signals (inset). The above process usually took about 10–15 s. Perfusion was resumed immediately after noticing a change in fluorescence signals. **(C)** Simplified situation of two hemispherical cells covered by a thin liquid film, where *T* is the surface tension of the liquid and ΔT is the net component of T, acting perpendicularly to the cell surface to cause cell deformation.

The approach to produce an ALI on urothelial cells is illustrated in Figure 1. The cell chamber was continuously perfused with Hank’s balanced salt solution (HBSS) containing (in mM): 138 NaCl; 5 KCl; 0.3 KH_2_PO_4_; 4 NaHCO_3_; 2 CaCl_2_; 1 MgCl_2_; 10 HEPES; and 5.6 glucose. The pH was adjusted with Tris base to 7.4, and osmolality was adjusted with sucrose to 320–325 mOsm. The influx of perfusion was driven by gravity, and the efflux was driven by a vacuum pump. Monolayer urothelial cells were immersed in HBSS when the influx and efflux flow rates were equally maintained, and no ALI was formed on the cells (Figure 1A).

When the influx was stopped but the efflux remained (vacuum was kept running), the amount of HBSS solution in the chamber was reduced to a level, at which a thin liquid layer was formed covering the cells. Cells were mechanically deformed by the tension produced at the ALI, and fluorescence signals at 340 and 380 nm were altered (inset of Figure 1B). The above process took about 10–15 s. The influx of perfusion was resumed immediately after noticing a change in the fluorescence signal. A simplified situation of two hemispherical cells covered by a thin liquid film, where *T* is the surface tension of the liquid and ΔT is a net component of *T*, acting perpendicularly to the cell surface to cause cell deformation (Figure 1C).

### Ca^2+^ Imaging

Urothelial cells were loaded with Fura 2-AM (2 μM, Dojindo, Japan) for 30–40 min at 37°C in an atmosphere of 95% O_2_ and 5% CO_2_. Fura 2-AM was dissolved in HBSS. Ca^2+^ imaging was performed as described previously (Zhang et al., 2011). Briefly, coverslips were placed under a fluorescence microscope (Nikon Eclipse Ti) and continuously perfused with HBSS (1.5–2 ml/min). Fura 2-AM was alternately excited with ultraviolet light at 340 and 380 nm. The fluorescence emission was detected at 510 nm using a computer-controlled monochromator. Wavelength selection, timing of excitation, and the acquisition of images were controlled using the MetaFluor software (Molecular Devices, Sunnyvale, CA, United States). Digital images were stored for offline analysis. The ratio of fluorescence signals measured at 340 nm divided by the fluorescence signals measured at 380 nm was used to determine the increase in intracellular Ca^2+^.

### ATP Assay

The ATP concentration was measured by using luciferin-luciferase bioluminescence. Samples (100 μl) of perfusate were collected 2 min before and immediately after ALI stimulation. A mixture of 100 μl luciferin-luciferase was added to a 100 μl sample, according to the manufacturer’s instructions using the Promega CellTiter-Glo Luminescent Cell Viability Assay Kit (Promega). ATP detection was evaluated using the GloMax 20/20 Luminometer (Promega). Sample bioluminescence was compared to that of standard amounts of ATP used within the same concentration range. All samples were run in duplicate.

### Immunofluorescence Staining

Immunofluorescence staining was performed to identify the expression of TRPV4 and pannexin 1 in urothelial cells. The bladder tissue was frozen in standard cryomolds containing optimal cutting temperature (OCT) compound. The tissue was then sectioned, permeabilized with 0.5% Triton X-100 for 30 min, and blocked with 5% goat serum for 1 h. The primary antibodies used were rabbit polyclonal anti-TRPV4 antibody (Abcam ab39260, 1:50); mouse monoclonal anti-cytokeratin AE1/AE3 antibody (Abcam ab86734, 1:100); rabbit polyclonal anti-pannexin 1 antibody (Abcam ab139715, 1:100), and normal rabbit IgG (Sigma 12–370, 1:100). The specificity of these antibodies has been determined in previous studies (Heppner et al., 2017; Chen et al., 2019). The tissue was then incubated with primary antibodies overnight at 4°C, washed three times with PBS, and incubated with secondary antibodies for 1 h. Secondary antibodies were goat anti-rabbit and mouse IgG FITC (1:250, Zhongshanjinqiao, China) and goat anti-mouse and mouse IgG 594 (1:250, Zhongshanjinqiao, China). The nuclei were counterstained with 4',6-diamidino-2-phenylindole (DAPI; Invitrogen).

### Statistical Analysis

All data were expressed as the mean ± SEM. Statistical significance was evaluated using the Student’s *t*-test or paired *t*-test, or one-way or two-way ANOVA with Bonferroni *post hoc* test for multiple comparisons, as appropriate. When the test for normality failed, the Mann-Whitney rank sum test was applied. Data were considered statistically significant when *p* < 0.05.

## Results

### ALI Evokes an Intracellular Ca^2+^ Increase and ATP Release in Urothelial Cells

**Figure 2:**
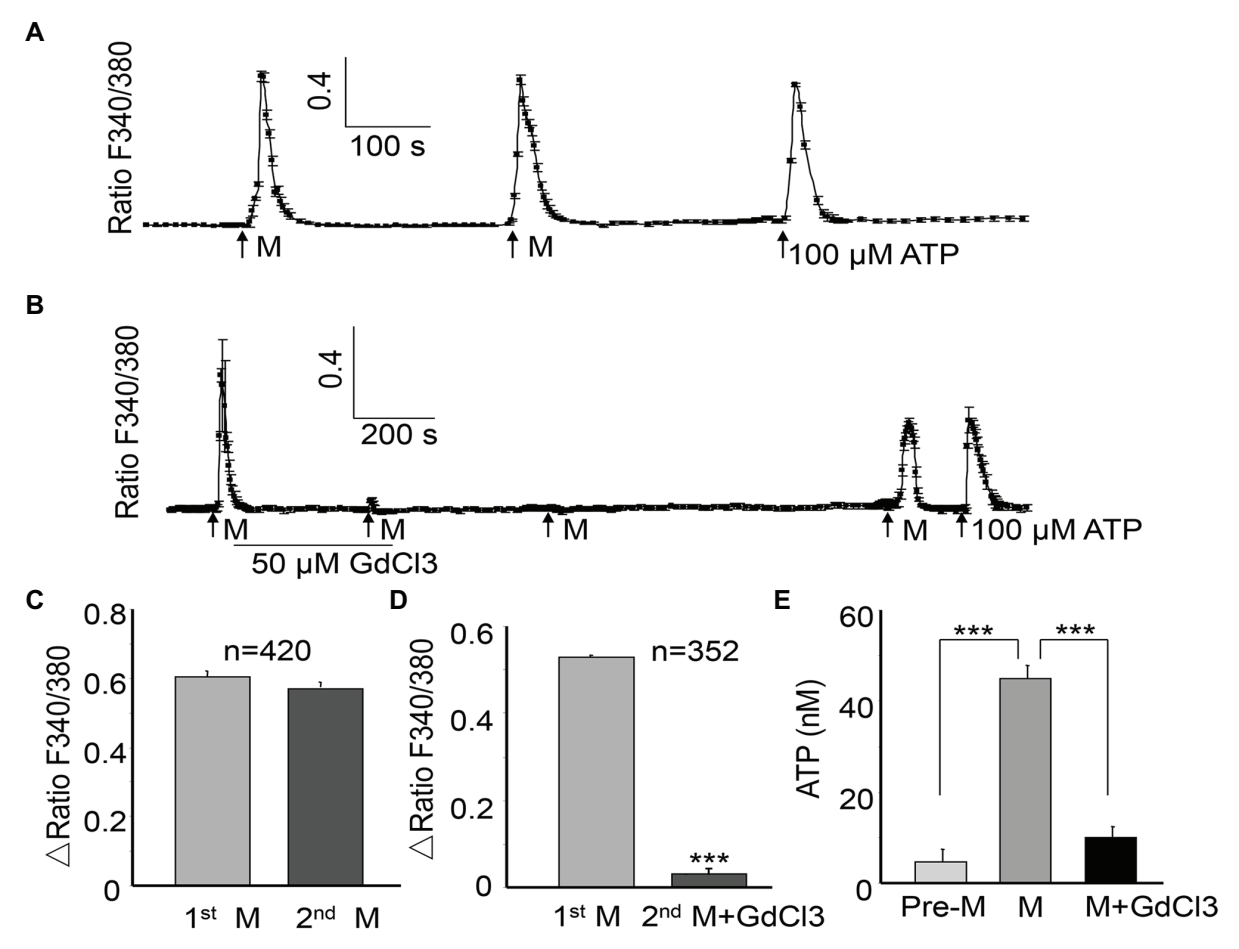
Air-liquid interface evokes the repeatable [Ca^2+^]_i_ increases and ATP release in urothelial cells, which can be blocked by GdCl3. A [Ca^2+^]_i_ increase is expressed as the ratio of fluorescence at 340 and 380 nm (F340/F380). **(A)** One typical trace showing the ALI (M)-evoked reversible [Ca^2+^]_i_ increase. The trace was obtained by averaging the responses of six cells from the same coverslip. The application of ATP (100 μM) at the end of the experiment evoked significant Ca^2+^ increase, indicating good viability of the cells. **(B)** One typical trace (averaging five cell responses) showing that GdCl3 (50 μM) almost abolished the ALI-induced [Ca^2+^]_i_ increase, and the blocking effect was long lasting. **(C)** Summary data from 420 cells showing that the amplitude of the second ALI-induced [Ca^2+^]_i_ increase was not significantly different from the first. The amplitude of the [Ca^2+^]_i_ increase was calculated by subtraction of the peak from baseline and expressed as Δ ratio of F340/F380. **(D)** Summary data from 352 cells showing that the amplitude of the [Ca^2+^]_i_ increase was significantly attenuated in the presence of GdCl3 (*p* < 0.001). **(E)** Among five coverslips, ALI evoked a significant increase in ATP concentration in the perfusate, compared with without ALI (Pre-M). Pretreatment with GdCl3 blocked the ALI-evoked ATP increase (*p* < 0.001). ATP concentration was measured as described in the Material and Methods section.

Several forms of mechanical stimuli, such as stretching (Miyamoto et al., 2014; Guan et al., 2018) and osmotic swelling (Haeberle et al., 2008; Wu et al., 2011) can trigger Ca^2+^ influx in urothelial cells. In the current study, ALI stimulation also evoked a remarkable increase in [Ca^2+^]_i_ in the cultured urothelial cells (Figure 2A). Moreover, this response was reversible (Figure 2A), and the second ALI-evoked increase in [Ca^2+^]_i_ showed no significant differences from the first stimulation (amplitude, 0.579 ± 0.017 for the first transient, vs. 0.546 ± 0.016 for the second transient, *n* = 420 cells, *p* > 0.05, Figure 2C). For the same coverslip, the application of ATP (100 μM, Sigma) at the end of the experiment evoked a significant increase in Ca^2+^ (Figure 2A), indicating that cells retained good viability after formation of the ALI. A significant increase in the ATP concentration of perfusates from five coverslips was observed after ALI stimulation (*p* < 0.001, Figure 2E). A model of two successive ALI stimulations with a 10–15 min interval (Figure 2A) was used in the following experiments to test the underlying mechanisms of the [Ca^2+^]_i_ increase.

### Mechanosensitive Channels Involved in the ALI-Evoked [Ca^2+^]_i_ Increase

The MSCs have been shown to be involved in tension-induced Ca^2+^ influx (Olsen et al., 2011). To determine the potential involvement of MSCs in the ALI-evoked [Ca^2+^]_i_ increase, GdCl_3_(Sigma), an unspecific MSC blocker, was applied between the two ALI. The GdCl_3_ (50 μM) treatment almost abolished the second ALI-evoked [Ca^2+^]_i_ increase (amplitude reduced by 94.30%, *p* < 0.001, Figures 2B,D). The inhibition produced by 50 μM GdCl_3_ was long lasting, and required 20–30 min washout for recovery of the ALI-induced response (Figure 2B). The ALI-evoked ATP release was also significantly reduced in the presence of GdCl_3_ (*p* < 0.001, Figure 2E).

### TRPV4 Activation Is Involved in the [Ca^2+^]_i_ Increase

**Figure 3:**
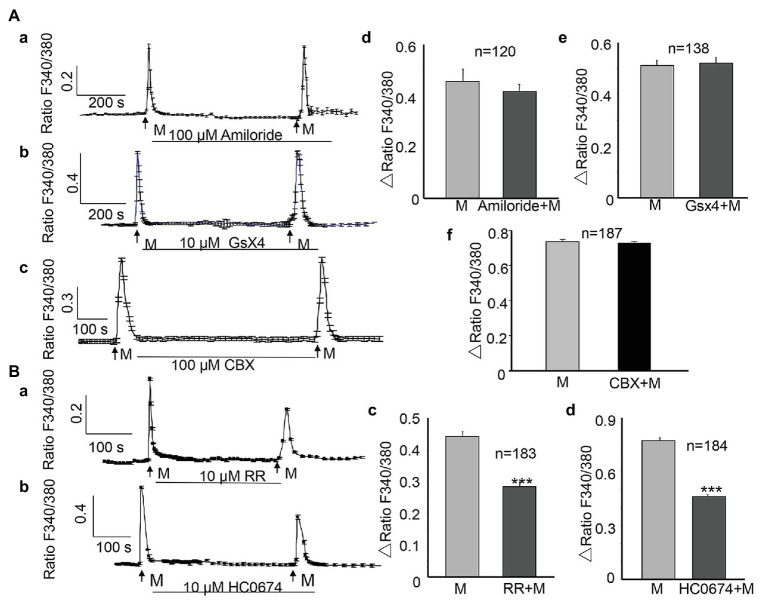
Activation of transient receptor potential vanilloid (TRPV4) channels but not EnaC, Piezo, and connexin channels contribute to the ALI-induced [Ca^2+^]_i_ increase. **(Aa–Ac)** Typical traces showing treatment with amiloride (100 μM), the ENaC channel blocker, GsMTX4 (GsX4, 10 μM), the Piezo channel antagonist, or carbenoxolone (CBX, 100 μM), the connexin channel blocker did not attenuate the [Ca^2+^]_i_ increase. **(Ad–Af)** Summary data for amiloride (**Ad**, *n* = 120 cells, *p* > 0.05) and GsX4 (**Ae**, *n* = 138 cells, *p* > 0.05), and CBX (**Af**, *n* = 187 cells, *p* > 0.05). **(Ba,Bb)** Typical traces showing treatment with ruthenium red (RR; 10 μM), the no-specific cation channel antagonist, and HC0674 (10 μM), the specific TRPV4 antagonist, reduced the ALI-induced [Ca^2+^]_i_ increase. **(Bc,Bd)** Summary data for RR (**Bc**, *n* = 183 cells, *p* < 0.001) and HC0674 (**Bd**, *n* = 184 cells, *p* < 0.001).

The epithelial Na^+^/degenerin (ENaC/DEG) channel (Du et al., 2007); Piezo 1 channel (Miyamoto et al., 2014); Piezo 2 channel (Marshall et al., 2020); connexin channel (Shimura et al., 2018), and TRPV4 channel (Mochizuki et al., 2009) are all MSCs that are reportedly present on urothelial cells. We examined the contribution of these MSCs to the ALI-induced [Ca^2+^]_i_ increase. The application of amiloride (100 μM, Sigma), the ENaC channel antagonist, or GsMTX4 (GsX4, 10 μM, MCE, China), one commonly used Piezo channel antagonist (Lee et al., 2014; Alcaino et al., 2017; Velasco-Estevez et al., 2020), or carbenoxolone (100 μM), a connexin channel blocker between the two successive inductions of the ALI and during the second ALI showed no significant inhibition (Figures 3Aa–Ac). No significant differences were observed in the amplitude between the first and the second ALI-induced [Ca^2+^]_i_ increases (*p* > 0.05, Figures 3Ad–Af), suggesting no involvement of the ENaC, Piezo, or connexin channels.

However, application of ruthenium red (RR, 10 μM, Sigma), a nonspecific inhibitor of many cation channels, including the TRP channels, significantly reduced the ALI-induced [Ca^2+^]_i_ increase (Figure 3Ba), and the amplitude of the second ALI-induced [Ca^2+^]_i_ increase was reduced by 35.7% (*n* = 183, *p* < 0.001, Figure 3Bc). The TRPV4 channels are highly expressed in the urothelium (Mochizuki et al., 2009). To evaluate the contribution of TRPV4 to the ALI-induced [Ca^2+^]_i_ increase, HC0674 (10 μM, Abcam), the specific TRPV4 antagonist, was applied between the two inductions of the ALI and during the second ALI. The amplitude of the second ALI-induced [Ca^2+^]_i_ increase was reduced by 38.8% (*n* = 184, *p* < 0.001, Figures 3Bb,Bd), compared with that of the first ALI-induced [Ca^2+^]_i_ increase.

### Source of the ALI-Evoked [Ca^2+^]_i_ Increase

**Figure 4:**
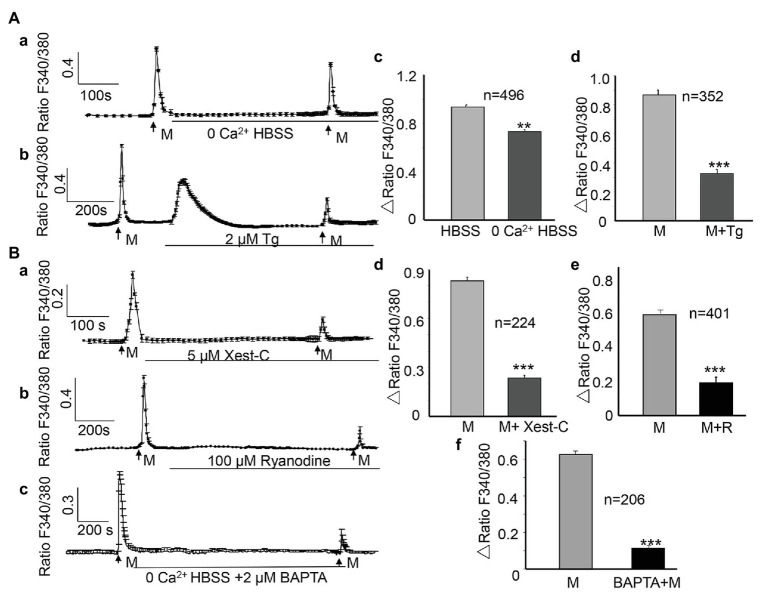
Both Ca^2+^ influx and Ca^2+^ release from intracellular stores contribute to the ALI-induced [Ca^2+^]_i_ increases. **(Aa,Ab)** Typical traces showing switching from normal HBSS to 0 Ca^2+^ HBSS **(Aa)** or depletion of intracellular Ca^2+^ stores with thapsigargin (**Ab**, Tg, 2 μM) reduced the ALI-evoked [Ca^2+^]_i_ increase. Note that thapsigargin alone induced a sustained increase of [Ca^2+^]_i_. **(Ac,Ad)** Summary data for 0 Ca^2+^ HBSS (**Ac**, *n* = 496 cells, *p* < 0.01); and thapsigargin (**Ad**, *n* = 352 cells, *p* < 0.001). **(Ba–Bc)** Typical tracings showing that Xest-C (5 μM), the IP3 receptor-specific antagonist **(Ba)**, ryanodine (100 μM), the ryanodine receptor antagonist **(Bb)**, and BAPTA-AM (2 μM, **Bc**) reduced the ALI-induced [Ca^2+^]_i_ increase. **(Bd–Bf)** Summary data for Xest-C (**Bd**, *n* = 224 cells, *p* < 0.001), ryanodine (**Be**, *n* = 401 cells, *p* < 0.01), and BAPTA-AM (**Bf**, *n* = 206 cells, *p* < 0.001).

To examine the contribution of Ca^2+^ influx from the extracellular space, cells were perfused with Ca^2+^-free HBSS, which was modified from normal HBSS through the omission of CaCl_2_, the addition of 0.1 mM EGTA, and an increase in the concentration of MgCl_2_ to 3.1 mM. The osmolality of Ca^2+^-free HBSS was adjusted with sucrose to 320–325 mOsm. In the presence of Ca^2+^-free HBSS, the ALI-induced [Ca^2+^]_i_ increase was significantly reduced by 22%, compared with that in the presence of normal HBSS (0.936 ± 0.018 vs. 0.731 ± 0.016, *n* = 496, *p* < 0.01 Figures 4Aa,Ac). To determine the involvement of Ca^2+^ release from the endoplasmic reticulum (ER) calcium stores, thapsigargin (Sigma), a blocker that irreversibly inhibits the sarcoplasmic/endoplasmic reticulum Ca^2+^-ATPase (SERCA) pumps that are responsible for calcium reuptake from the cytoplasm back into the ER, was applied between the two ALI stimuli and during the second ALI stimulus. The second ALI-induced [Ca^2+^]_i_ increase was significantly reduced by 62.4% in the presence of thapsigargin (2 μM; 0.867 ± 0.034 vs. 0.326 ± 0.029, *p* < 0.001, Figures 4Ab,Ad). Notably, thapsigargin alone evoked a remarkable [Ca^2+^]_i_ increase (Figure 4Ab).

### Activation of IP3 and Ryanodine Receptors on the ER Contribute to the [Ca^2+^]_i_ Increase

The ryanodine receptor (RyR) and inositol 1,4,5-trisphosphate receptor (IP3R; Galione and Churchill, 2002) are the two major Ca^2+^-release receptor channels of the ER. To determine whether these two receptors contribute to the ALI-induced [Ca^2+^]_i_ increase, xestospongin-C (Xest-C; Mijares et al., 2020; Sigma), the IP3R specific antagonist, or ryanodine (MCE, China), the RyR antagonist, were applied between the two ALI stimuli and during the second ALI stimulus. Both Xest-C (5 μM) and ryanodine (100 μM) significantly reduced the second ALI-induced [Ca^2+^]_i_ increase (Figures 4Ba,Bb), and the amplitude was decreased by 71.6 and 67.7%, respectively (Figures 4Bd,Be). To further examine the contribution of intracellular Ca^2+^ release, BAPTA-AM (2 μM), a membrane-permeable form of BAPTA was applied between the two ALI stimuli and during the second stimulus to clamp intracellular Ca^2+^ levels (Birder et al., 2003). Second ALI induced [Ca^2+^]_i_ was significantly reduced (Figures 4Bc,Bf).

### Activation of the Pannexin 1 Channel Is Involved in the ALI-Induced [Ca^2+^]_i_ Increase and ATP Release

**Figure 5:**
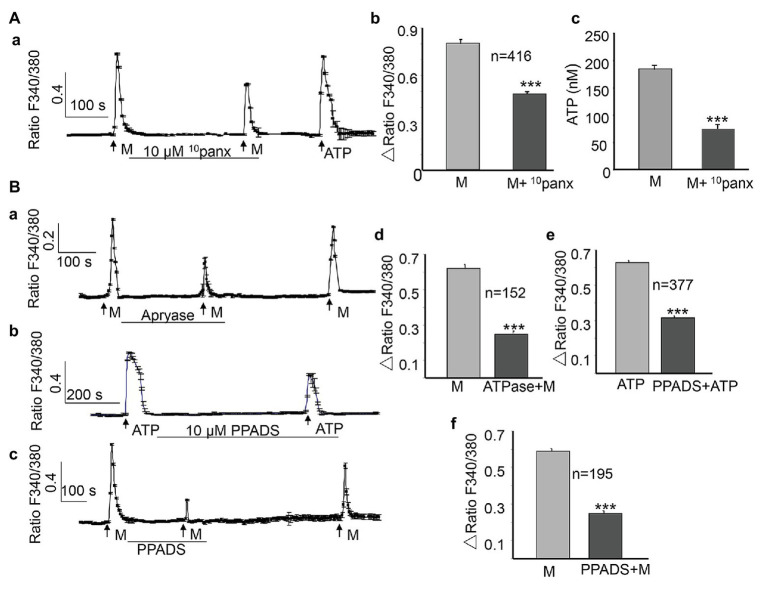
Activation of pannexin 1 channels and ATP release contribute to the ALI-induced [Ca^2+^]_i_ increase. **(Aa–Ac)** Typical trace **(Aa)** and summary data (**Ab**, *n* = 416 cells, *p* < 0.001) showing that the application of ^10^panx (10 μM), the pannexin 1 channel-specific antagonist, reduced the ALI-induced [Ca^2+^]_i_ increase and ALI-induced ATP release **(Ac)**. **(Ba–Bc)** Typical traces showing that the application of apyrase (10 U/ml) to hydrolyze extracellular ATP reduced ALI-evoked [Ca^2+^]_i_ increase **(Ba)**; and the application of PPADS (10 μM), the common P2 receptor antagonist, reduced ATP evoked **(Bb)**, as well as the ALI-evoked [Ca^2+^]_i_ increase **(Bc)**. **(Bd–Bf)** Summary data for apyrase effect (**Bd**, *n* = 152 cells, *p* < 0.001); PPADS effect on ATP evoked [Ca^2+^]_i_ increase (**Be**, *n* = 377 cells, *p* < 0.001); and on the ALI-evoked [Ca^2+^]_i_ increase (**Bf**, *n* = 195 cells, *p* < 0.001).

Pannexin 1 channels play essential roles in urothelial mechanotransduction (Negoro et al., 2014). Besides its mechanical sensitivity, a nonvesicular mechanism of ATP release, mediated by pannexin channels in urothelial cells, has been identified in recent years (Penuela et al., 2013). To determine whether pannexin 1 channels are involved in the ALI-induced [Ca^2+^]_i_ increase and release of ATP, ^10^panx (Burma et al., 2017), the pannexin 1 channel peptide blocker, was applied between the two ALI stimuli. The application of ^10^panx (10 μM) significantly reduced the second ALI-induced [Ca^2+^]_i_ increase by 39.3% (*p* < 0.001, Figures 5Aa,Ab), as well as the amount of ATP release (*p* < 0.001, Figure 5Ac).

### Role of Autocrine ATP in the ALI-Induced [Ca^2+^]_i_ Increase

In urothelial cells, ATP can induce a [Ca^2+^]_i_ increase by activating P2X or P2Y receptors (Locovei et al., 2007; von Kugelgen and Hoffmann, 2016). The autocrine ATP signaling in urothelial cells contributes to a major part of the mechanical-mediated [Ca^2+^]_i_ increase (Guan et al., 2018). To investigate whether the ALI-induced ATP release could lead to a [Ca^2+^]_i_ increase in an autocrine or paracrine manner, we first applied the ATP-diphosphohydrolase (apyrase, from Sigma) to degrade extracellular ATP by catalyzing its breakdown to ADP. As shown in Figure 5B, the application of apyrase (10 U/ml) significantly blocked the ALI-induced [Ca^2+^]_i_ increase by 59.7% (*n* = 152, *p* < 0.001, Figures 5Ba,Bd), and this blocking effect was reversible. To verify the role of P2X and P2Y purinergic receptors, we evaluated the effects of pyridoxalphosphate-6-azophenyl-2',4'-disulfonic acid (PPADS; MCE, China), the nonselective purinergic receptor blocker. As expected, the application of PPADS (10 μM) significantly inhibited the ATP-evoked [Ca^2+^]_i_ increase (Figures 5Bb,Be, *p* < 0.001). Furthermore, PPADS (10 μM) significantly blocked the ALI-induced [Ca^2+^]_i_ increase by 49.7% (Figures 5Bc,Bf), and these effects were reversible.

### TRPV4 And Pannexin-1 Expression in the Urothelium

**Figure 6:**
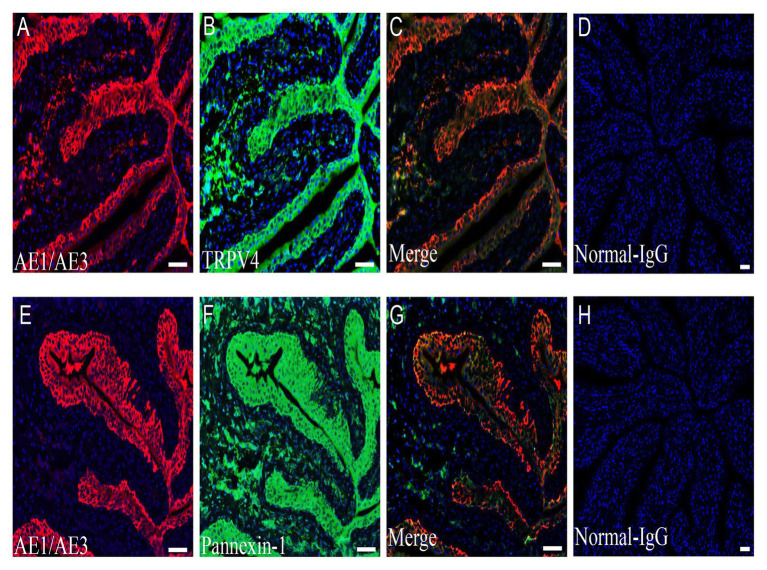
Expression of TRPV4 and pannexin 1 channel on the rat bladder urothelium. **(A–C)** Urothelial cell marker AE1/AE3-IR (red, **A**); TRPV4-IR (green, **B**); and double staining (yellow, **C**) in the urothelial layer. **(E–G)** Urothelial cell marker AE1/AE3-IR (red, **E**); pannexin 1-IR (green, **F**); and double staining (yellow, **G**) in the urothelial layer. **(D,H)** Image with normal rabbit IgG. Blue color in all panels represents nuclear staining by 4',6-diamidino-2-phenylindole (DAPI). TRPV4-IR and pannexin 1-IR span from the basal layer to umbrella cells. All panels are shown at the same magnification. Scale bars represent 100 μm.

Immunofluorescence staining for pannexin 1 and TRPV4 channels revealed strong staining in cells adjacent to the lumen of the bladder (Figures 6B,F). Colocalization studies with AE1/AE3, a molecular marker of urothelial cells, confirmed that pannexin 1 and TRPV4 are both expressed in the urothelium (Figures 6C,G). Negative control experiments with normal rabbit IgG showed no staining (Figures 6D,H).

## Discussion

In the current study, the cellular mechanisms underlying ALI-induced increase in [Ca^2+^]_i_ of cultured urothelial cells were investigated using various pharmacological blockers. Our main findings were as follows: (1) Both Ca^2+^ influx and Ca^2+^ release from intracellular Ca^2+^ stores contribute to the [Ca^2+^]_i_ increase; (2) TRPV4 or pannexin 1 channels are the two membrane-bound MSCs involved in Ca^2+^ influx; (3) activation of both IP3 and ryanodine receptors on the ER contribute to Ca^2+^ release from intracellular Ca^2+^ stores; and (4) ATP release also contributes to the [Ca^2+^]_i_ increases by activating P2X and P2Y receptors *via* autocrine or paracrine mechanisms.

*In vitro*, commonly used approaches to deliver mechanical stimulation to urothelial cells include: stretching the cells with various custom designed devices (Miyamoto et al., 2014; Guan et al., 2018); exposing the cells to high hydrostatic pressure; or simply subjecting the cells to a hypoosmotic shock (Olsen et al., 2011). Using these approaches, valuable information has been obtained regarding urothelial cell mechanotransduction. For example, TRPV4 or Piezo1 channels have been revealed as the MSCs responsible for sensing mechanical stimuli (Mochizuki et al., 2009; Miyamoto et al., 2014). In addition, the activation of pannexin 1 channels has been considered an important mechanism of ATP release (Penuela et al., 2013). However, the above approaches usually require technically complicated setups.

In the current study, another form of mechanical stimulation was established, i.e., creation of an ALI on urothelial cells. The ALI evoked similar cellular responses in urothelial cells as the mechanical modalities mentioned above, i.e., [Ca^2+^]_i_ increase and ATP release. Moreover, the whole procedure did not cause cell lysis, which was evidenced by the remarkable [Ca^2+^]_i_ increase in response to ATP after inductions of ALI. Thus, an ALI can induce the [Ca^2+^]_i_ increase and ATP release, but does not necessarily cause damage to the plasma membrane. Although the chances that the bladder urothelium is exposed to an ALI are not common under physiological conditions, this may occur during some invasive procedures, like bladder surgeries under laparoscopy or during cystoscopy.

An ALI may also be frequently encountered in urothelial cell culture experiments. For example, when the culture medium is changed. In the current study, we did not observe to what extent cell deformation was evoked by the ALI. When the cell is exposed to an ALI, surface tension forces up to 320 Newtons/in^2^ could be exerted on the cell membrane (Ludwig et al., 2008), and the pressure generated by surface tension is large enough to cause significant cell deformation. Thus, we established a simple procedure to deliver mechanical stimulation to cultured urothelial cells, which can be used as a valuable model to investigate the cellular mechanisms of [Ca^2+^]_i_ increase or ATP release.

Tension-induced Ca^2+^ entry into cells is reportedly achieved through the activation of MSCs (Olsen et al., 2011; Leybaert and Sanderson, 2012; Jafarnejad et al., 2015). In the current study, the involvement of MSC channels in the ALI-induced [Ca^2+^]_i_ increase could be supported by the observation of the almost complete blockade of the ALI-induced [Ca^2+^]_i_ increase by GdCl_3_. As a common nonspecific MSC blocker, Gd^3+^ has been shown to block many MSC channels, including several types of TRP channels (White et al., 2016), pannexin 1 channels (Jiang and Penuela, 2016), as well as mechano-insensitive channels like those associated with purinergic receptors (Khakh et al., 2001).

In previous studies, TRPV4 (Mochizuki et al., 2009), epithelial Na^+^ (ENa; Du et al., 2007), Piezo1 (Miyamoto et al., 2014), Piezo 2 (Marshall et al., 2020), connexin (Shimura et al., 2018), and pannexin 1 channels (Timoteo et al., 2014; McLatchie and Fry, 2015) have been implicated in mechanotransduction of urothelial cells. In agreement with these previous reports, our observations of the significant reduction of the [Ca^2+^]_i_ increase by RR, as well as the highly TRPV4-selective antagonist, suggest the involvement of TRPV4 channels. The expression of TRPV4 in the urothelium further supports the contribution of TRPV4. However, our observations that the ENaC antagonist (amiloride), the Piezo channel antagonist (GsMTx4), and the connexin channel antagonist (carbenoxolone) could not block the ALI-induced [Ca^2+^]_i_ increase invalidates the involvement of these MSCs.

These results are in contrast with those of previous reports, which state that Piezo1, Piezo2 (Marshall et al., 2020), ENaC, or connexin channels (Shimura et al., 2018) contribute to mechanosensory transduction of the bladder urothelium (Du et al., 2007; Miyamoto et al., 2014). These inconsistent results may be attributed to the different ways in which the cells were stimulated. In the study Miyamoto et al. (2014), mouse urothelial cells were horizontally elongated by extending the cell chamber. In the current study, the apical surface of urothelial cells were exposed to the ALI, such that any curved cell surface was subjected to a net component of tension forces that acted perpendicularly to the cell membrane (Figure 1B). Thus, the inconsistency in results may imply that urothelial cells use different mechanosensing channels in response to various forms of tension forces (horizontal vs. perpendicular).

Pannexin 1 channels are characterized by their mechanosensitivity and their ability to form large pores that are permeable to ATP and other molecules (Penuela et al., 2013). Therefore, they are reportedly involved in the nonvesicular release of ATP in the bladder urothelium, in response to mechanical stimulation (McLatchie and Fry, 2015). Consistent with this theory, our observation that ^10^panx, the specific pannexin 1 channel antagonist, significantly attenuated both the ALI-induced [Ca^2+^]_i_ increase (by 39.3%) and ATP release indicates the involvement of pannexin 1 channels. Furthermore, our immunohistochemical analysis demonstrated the expression of the pannexin 1 channel in urothelial cells.

We found that the ALI-induced [Ca^2+^]_i_ increase was reduced by 22% in Ca^2+^-free HBSS and was reduced by 62.4% following the application of thapsigargin, a blocker that irreversibly inhibits the ER Ca^2+^-ATPase pumps. These results suggest that compared with Ca^2+^ entry, Ca^2+^ release from the ER calcium stores are the dominant source of the ALI-induced [Ca^2+^]_i_ increase. The contribution of Ca^2+^ release from the ER calcium stores can also be supported by the observation that ALI induced [Ca^2+^]_i_ was significantly reduced by application of BAPTA-AM to clamp intracellular Ca^2+^ levels (Figure 4Bc). The IP3 receptor (IP3R) and ryanodine receptor on the ER have been implicated in Ca^2+^ release from intracellular calcium stores, and play an important role in Ca^2+^-induced Ca^2+^ release (CICR; Galione and Churchill, 2002). In agreement with this idea, when the ER calcium stores were blocked with the antagonist, IP3R (Xest-C), or the ryanodine receptor, the ALI-induced [Ca^2+^]_i_ increase was reduced by 71.6 and 67.7%, respectively. The CICR was mediated by the activation of ryanodine receptors located on the ER, which can be blocked by high concentrations of ryanodine (100 μM; Bouchard et al., 2003). In the current study, the blocking effects of Xest-C and ryanodine may imply the involvement of the CICR mechanism in the ALI-induced [Ca^2+^]_i_ increase.

One report on a human urothelial cell line (T24 cells) showed that ATP released upon cell stretching contributes to a major part of Ca^2+^ signaling (Guan et al., 2018). In agreement with this finding, we also observed that ALI-evoked ATP release contributed to the ALI-induced [Ca^2+^]_i_ increase *via* paracrine or autocrine mechanisms. This conclusion can be supported by the following evidence: (1) the ALI-induced [Ca^2+^]_i_ increase was significantly reduced by ATP hydrolysis with apyrase and (2) the [Ca^2+^]_i_ increase was significantly reduced by blocking the P2X and P2Y purinergic receptors with PPADS. Thus, autocrine ATP signaling works like an amplifier to increase the concentration of cytosolic Ca^2+^. We did not examine which type of purinergic receptors is involved with these processes. However, previous studies have shown that the P2X receptor that mediates the increase in [Ca^2+^]_i_ is dependent on extracellular Ca^2+^. On the other hand, P2Y receptors are G-protein-coupled receptors that can induce the release of Ca^2+^ from intracellular stores *via* the IP3 pathway (von Kugelgen and Hoffmann, 2016). Based on the dominant role of intracellular Ca^2+^ release from the ER in the ALI-induced [Ca^2+^]_i_ increase, also based on our observation that the amplitude of ATP evoked [Ca^2+^]_i_ increase in zero Ca^2+^ bath is not significantly different from that in normal HBSS bath (data not shown), we propose that P2Y receptors may have a greater contribution to the process than P2X receptors.

**Figure 7:**
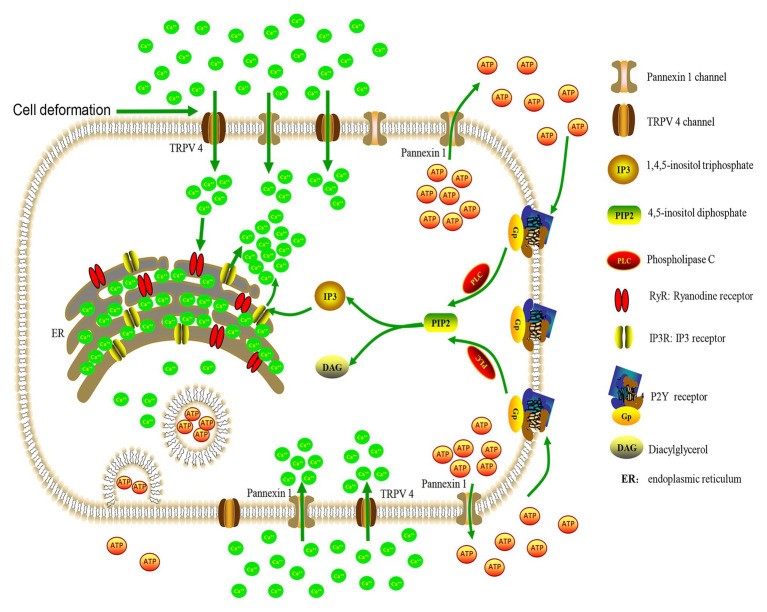
A working model of cellular mechanisms for ALI-induced a [Ca^2+^]_i_ increase. Cell deformation activates membrane-bound TRPV4 or pannexin 1 channels to cause Ca^2+^ influx and [Ca^2+^]_i_ increase, which in turn activates IP3 or ryanodine receptors on the endoplasmic reticulum (ER) to induce Ca^2+^ release from intracellular Ca^2+^ stores to further increase the [Ca^2+^]_i_ levels. The activation of pannexin 1 channels or the [Ca^2+^]_i_ increase can lead to ATP release, through nonvesicular or vesicular mechanisms, respectively. ATP further enhances the [Ca^2+^]_i_ increase by acting on P2Y receptors to increase IP3 level and Ca^2+^ release from Ca^2+^ stores.

Based on the current results, a working model has been proposed (Figure 7). The process is likely initiated with the activation of TRPV4 or pannexin 1 channels and Ca^2+^ influx, which in turn activates IP3 or ryanodine receptors on the ER to induce Ca^2+^ release from intracellular Ca^2+^ stores to further increase [Ca^2+^]_i_. Activation of the pannexin 1 channels or [Ca^2+^]_i_ increase leads to ATP release through nonvesicular or vesicular mechanisms, respectively, which further enhances the increase in [Ca^2+^]_i_ by activating at P2X and P2Y receptors *via* autocrine or paracrine mechanisms.

Notably, the method used to create the ALI-induced plasma membrane deformation by causing changes in the perfusion volume may alter the equilibrium and concentration of signaling molecules close to the plasma membrane, thereby altering their autocrine/paracrine effects. Typically, this is the case with the ATP concentration; transient reduction of the perfusion fluid volume may increase the concentration of the nucleotide. Compared with other techniques, this is a disadvantage of the ALI method to induce urothelial cell deformation and is a limitation of the current study. Another limitation is the pharmacological approach employed for most of our experiments to determine the associated channels or receptors. Some of the antagonists used in the current study may not be very specific.

## Conclusion

In summary, we revealed the cellular mechanisms underlying membrane tension force induced [Ca^2+^]_i_ increase in urothelial cells. Changes of any one of the associated channels or receptors (Figure 7) may affect bladder mechanotransduction and sensory function. Although the chances that the bladder urothelium exposed to an ALI is uncommon under physiological conditions, detrusor overactivity that occurs in overactive bladder conditions can increase deformation in the urothelium, which could trigger an increase in [Ca^2+^]_i_ and the release of ATP, independent of urine volume.

## Data Availability Statement

The original contributions presented in the study are included in the article/supplementary material, further inquiries can be directed to the corresponding author.

## Ethics Statement

The animal study was reviewed and approved by the Ethics Committee of the Second Hospital, Cheeloo College of Medicine, Shandong University [KYLL-2016(GJ)A-0027].

## Author Contributions

XZ and SW participated in research design. JW, ZC, SW, and MZ conducted experiments. JW, XZ, SZu, ZC, and MZ performed data analysis. XZ, SZh, and JW wrote the manuscript. All authors contributed to the article and approved the submitted version.

### Conflict of Interest

The authors declare that the research was conducted in the absence of any commercial or financial relationships that could be construed as a potential conflict of interest.
